# Effect of Nitrous Oxide Gas

**Published:** 1873-06

**Authors:** 


### ARTICLE V.

### *Effect of Nitrous Oxide Gas.*

In consequence of two or three deaths recently from the influences of nitrous oxide gas, an active discussion has been going on in France and England upon the subject, and various persons have been giving their experiences. One gentleman, a licentiate in dental surgery, thus narrates the effect of inhalation had upon him. The account is taken from the *British Journal of Dental Science.* He says:—

"I had to undergo a painful operation. I wished to take the nitrous oxide gas. I had administered it a great number of times, and in hearing the incoherent way in which patients expressed themselves as to the sensation whilst passing into an anæsthetic state, I hoped I might be able to define somewhat the effects of the gas. I say I hoped to be able, for of course, it is impossible to say what may be the effect of such an action upon any individual until we have experienced it, as I have no doubt different persons are differently affected, and the gas is so interesting and important to us that everything concerning it is worthy of notice.

I was well attended. Two Fellows of the Royal College of Surgeons and the anæsthetist—all were standing behind me, so that I could only see the face of the anæsthetist as he leaned over my shoulder with the mouthpiece; the other two were quite hidden from me. I am quite sure of that; I was perfectly composed, and as soon as the pipe was put to my face I formed a resolution steadily to take in the gas. My eyes were open and I looked at the distant wall. I heard them say, 'He takes in the gas freely,' which were the last words I heard distinctly; then I felt my eyes droop and close. Now I seemed to be in a different atmosphere, just as we feel in passing into a tropical house at Kew Gardens—different, but not an unpleasant atmosphere. As to choking we hear so much about, or soffocation, I felt not even the least repugnance. The only effect was, I remember thinking to myself this is another atmosphere; it seemed sweet and sooty; at the same time my ears were filled with a burring sound, such as I suppose is felt in descending the diving-bell, but not so violent. Immediately there danced before me a violent light the size of a large candle, and with a strange unearthly motion it flickered, rising up higher and higher, and I seemed to be strangely upborne with it. Up, up we went to a very high altitude, the burring sound always present. At last the light stood still, the burring ceased, and my attention was simply fixed on the light. It seemed an immense height we had come. To this stage I seemed to be a nonentity all my care had been devoted to the sound in my ears and the movement of the light; all the unpleasantness of the atmosphere had passed away. Now, however, a change came over me: became a person—a some one—and I felt as though I could see from every part of me —a sort of cataleptic state; and just as in looking over the cliff at Beechy Head on a calm day you hear conversation on the sea below, though you cannot discern the men in the skiff, so now in a strange muttering undertone I heard a voice as though explaining something about *me* to others. I was sure there were others present, and though explaining something I did not know a single word of what was said, and gradually there crept over me the conviction that I was bound helplessly, and they were doing something to me. There was a dead calm; the mutter ceased; I could see them looking intently with heads inclined. Simultaneously they raised their heads and the voice again spoke, but I had no pain, nor had I any pain or feeling when the gash was made; indeed (though I was cut in two places) not even the slightest prick; still I knew when the tumor was punctured. Although it was awfully tender to the slightest touch in my normal state, I had not the slightest pain until the tumor was violently pressed to force all the blood out; then I was conscious of painful sensation, and I groaned, as I thought, on account of the severity of the pain from squeezing. I felt whatever they were doing to me was now done, and done successfully, and I wanted to express my thankfulness, but I found myself unable to move or speak. Now the burring commenced, and the light, which had hung all the time shining over my head, began to descend, and I with it, and gradually the talking became nearer and more audible, the light went out, and the burring sound died away in the distane, and my eyes opened and with a heart full of gratitude I stretched out my hand to the gentleman and cried out lustily—'Thank God! thank God!' To which they replied—'It is all right; it is nicely done.' I rejoined—'I know it is. I know all about it.'

"I asked if I had made any groaning when the pressure was applied, and I was surprised to hear that I was not only silent but also motionless all the time. I took four gallons of gas, and from putting on the facepiece to my regaining consciousness was seventy seconds.

"I was not conscious of any sensation or nausea or giddiness, but a decided consciousness of disturbance, sensation of pricking about the cardiac region, and great pain in the tibiae, like a severe rheumatic pain, which increased, and though in bed, it settled into a dead coldness and want of circulation, which was not removed till I took two stiff glasses of stimulants; since that I have had no inconvenience." *Druggist's Circular.*

---

